# Time since last birth and the risk of endometrial cancer: A meta-analysis of observational studies

**DOI:** 10.1371/journal.pone.0325907

**Published:** 2025-07-08

**Authors:** Juan Gu, Yuchen Lai, Huafeng Shou, Liping Wang

**Affiliations:** 1 Department of Gynecology, Zhejiang Provincial People’s Hospital and People’s Hospital of Hangzhou Medical College, No. 158 Shangtang Road, Hangzhou, Zhejiang, China; 2 Center for General Practice Medicine, Zhejiang Provincial People’s Hospital and People’s Hospital of Hangzhou Medical College, No. 158 Shangtang Road, Hangzhou, Zhejiang, China; 3 Department of nursing, Zhejiang Provincial People’s Hospital and People’s Hospital of Hangzhou Medical College, No. 158 Shangtang Road, Hangzhou, Zhejiang, China; Imperial College London, UNITED KINGDOM OF GREAT BRITAIN AND NORTHERN IRELAND

## Abstract

**Introduction:**

Endometrial cancer, an adenocarcinoma originating from the uterine lining, is the most prevalent cancer of the female genital tract globally.Identifying early risk factors for endometrial cancer is crucial for prevention.Prior research suggests that pregnancy may lower endometrial cancer risk by reducing estrogen exposure.This meta-analysis aims to delve into the existing population-based longitudinal studies to evaluate the association between the time elapsed since the last birth and the risk of endometrial cancer.

**Methods and analysis:**

We searched PubMed, Cochrane Library, Embase, and Web of Science for cohort studies published up to June 21, 2024, using relevant medical subject headings (MeSH) and keywords. Statistical analyses were conducted using Stata version 14.0. A fixed-effects model was applied if P > 0.1 and I^2^ ≤ 50%; otherwise, a random-effects model was used to account for significant heterogeneity Publication bias was assessed using funnel plots and Egger’s test. Our meta-analysis included 3 cohort studies and 5 case-control studies with a total of 3,310,734 participants, published between 1994 and 2024. The analysis revealed that time since last birth is associated with endometrial cancer risk. Specifically, a period of 0–10 years since the last birth was linked to a reduced risk of endometrial cancer (OR= 0.431; 95% CI: 0.351–0.530). A period of 10–20 years since the last birth also showed a decreased risk (OR=0.867; 95% CI:0.747–1.007), whereas more than 20 years since the last birth was associated with an increased risk (OR = 1.304; 95% CI: 1.111–1.530).

**Conclusions:**

Our meta-analysis indicates that a shorter time since the last birth is protective against endometrial cancer, whereas a longer interval increases risk. Further research is needed to clarify the underlying mechanisms of this association. These findings are crucial for developing new strategies for endometrial cancer prevention and treatment.

**Prospero registration number**

International Prospective Register of Systematic Reviews (PROSPERO)

CRD42025644114.

## Introduction

Endometrial cancer, an adenocarcinoma originating from the uterine lining, is the most prevalent cancer of the female genital tract globally, with its incidence rising steadily [1,2]. Despite significant advances in disease prevention research, progress in therapeutic interventions remains limited, and clinicians are still awaiting effective disease-modifying treatments. Identifying early risk factors for endometrial cancer is crucial for prevention. Although several risk factors such as polycystic ovarian syndrome (PCOS), anovulatory infertility, age, family history, tamoxifen use, high BMI, and diabetes have been well-studied, pregnancy-related factors have received less attention [3–5].

Pregnancy impacts the endometrium through notable changes, including an increase in glandular and vascular development to form the placenta, which supplies the embryo and fetus with essential nutrients [6]. Prior research suggests that pregnancy may lower endometrial cancer risk by reducing estrogen exposure [7]. However, it is important to note that other pregnancy-related variables, such as whether a woman has ever been pregnant, the number of pregnancies (parity), and the patient’s age during pregnancies or at the time of diagnosis, may also influence endometrial cancer risk and serve as potential confounders. While this study focuses on time since last birth, further investigation of these variables is warranted [7,8].Some pregnancy-related factors, like placental growth factor (PlGF) and placenta-specific protein 1 (PLAC-1), have been linked to endometrial cancer [9–13], and associations with pregnancy-related Wnt signaling or Homeobox (HOX) genes have also been observed [2,14,15].

Despite evidence indicating pregnancy’s protective effect against endometrial cancer, the specifics of this protection, particularly the influence of the time since last birth, remain unclear. This meta-analysis aims to delve into the existing population-based longitudinal studies to elucidate the relationship between time since last birth and the risk of endometrial cancer, with the ultimate goal of clarifying how this factor may contribute to cancer risk.

## Methods

This meta-analysis adhered to the Preferred Reporting Items for Systematic Reviews and Meta-Analyses (PRISMA) guidelines [16]. The study protocol was pre-registered with the International Prospective Register of Systematic Reviews (PROSPERO)(CRD42025644114).

### Data sources

We conducted a systematic search of PubMed, Cochrane Library, Embase, and Web of Science from their inception to May 15, 2024. The search was not restricted by language. For non-English studies, initial translations were performed using professional translation software, followed by verification and review by bilingual members of the research team to ensure accuracy. We employed a search strategy that combined both medical subject headings (MeSH) and keywords related to the topic. The search terms included “Birth,” “Pregnancy,” “Gestation,” “Endometrial Neoplasms,” “Cancer of the Endometrium,” “Carcinoma of Endometrium,” and “Cancer of Endometrium.” The detailed search strategy is provided in S1 File. Additionally, we examined reference lists of the included studies and relevant meta-analyses to identify any further relevant trials.

### Eligibility criteria

We included studies based on the following criteria:

1) Cohort studies or case-control studies that explored the association between the time since last birth and the risk of endometrial cancer. These study designs were selected because they allow for a clearer understanding of temporal and causal relationships, whereas cross-sectional studies were excluded due to their inability to establish temporality.
2) Studies that reported odds ratios (ORs) with corresponding 95% confidence intervals (CIs). Studies that did not report these measures were excluded from the meta-analysis but were reviewed to ensure no critical information was missed for the systematic review.

We excluded conference abstracts, study protocols, duplicate publications, and studies without relevant outcome data. When multiple reports from the same cohort were identified, we prioritized studies with the longest follow-up or the largest sample size.

### Study selection

Two reviewers (GJ and WLP) independently screened the literature according to the predefined eligibility and exclusion criteria. Initially, articles were screened for relevance based on titles and abstracts, resulting in the exclusion of duplicates and irrelevant studies. Subsequently, full texts of potentially eligible articles were retrieved and assessed to determine their suitability for inclusion. Any disagreements were resolved through consultation with a third reviewer (LYC), who acted as an arbiter.

### Data extraction

Data extraction was carried out independently by reviewers GJ and WLP using pre-designed forms. The extracted information included the first author, publication year, study design, sample size, participant demographics (such as age), diagnosis of endometrial cancer, and adjusted confounders. While most studies adjusted for key confounders such as age and BMI, not all studies included these adjustments, which could introduce residual confounding. Discrepancies in data extraction were resolved through discussion with LYC to reach consensus.

### Risk of bias

The quality of the included cohort or case-control studies was evaluated using the Newcastle-Ottawa Scale (NOS). The NOS assigns a maximum of 9 stars, distributed as follows: four stars for selection and exposure measurement, two stars for comparability, and three stars for outcome assessment and follow-up. Studies were categorized as low, moderate, or high quality based on their total scores of 0–3, 4–6, and 7–9 stars, respectively. Studies with a high risk of bias were not excluded but were assessed in sensitivity analyses to determine their impact on the overall results. The Newcastle-Ottawa Scale scores indicated that all included studies were of moderate to high quality, with a mean score of 7.5.

### Statistical analysis

Adjusted odds ratios (ORs) and their corresponding 95% confidence intervals (CIs) were used to assess the relationship between the time since last birth and endometrial cancer risk. Heterogeneity among studies was evaluated using the χ² test and the I² statistic. A fixed-effects model was applied when P > 0.1 and I² ≤ 50%; otherwise, a random-effects model was used if I² > 50%, indicating substantial heterogeneity. Sensitivity analyses were performed by sequentially excluding individual studies to test the robustness of the overall results. Publication bias was assessed both visually using funnel plots and statistically with Egger’s regression test. Subgroup analyses were carried out based on study type and gender. All statistical analyses were performed using Stata statistical software version 14.0 (Stata Corp, College Station, Texas) [17,18].

## Results

### Literature search

**Fig 1:**
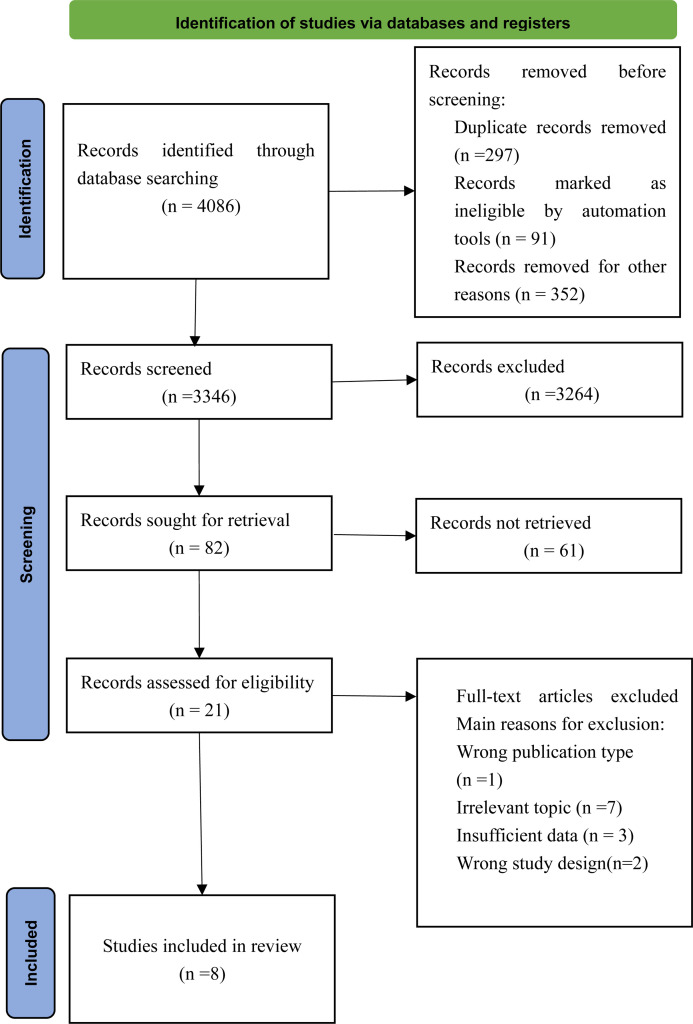
Study selection process.

The systematic search of cohort studies published before June 21st, 2024, identified 4086 results. After title and abstract screening, 8 articles were considered potentially relevant. Eight studies [19–26] were included after full text review, of which 8 reported the incidence of endometrial cancer on follow-up. The inclusion process explicitly excluded studies focusing solely on nulliparous women, as the research primarily examines the effect of time since last birth rather than parity status. The selection process is presented in Fig 1.

### Study characteristics

This meta-analysis included 5 case-control studies and 3 cohort studies covering 3,310,734 individuals, which were published between 1995 and 2024. The adjusted estimates were available for almost all studies even though the adjusted confounders are slightly different. The main characteristics of the included trials are shown in Table 1.

**Table 1:** Basic characteristics included in the study.

Author	Year	Country	Study Type	Diagnostic criteria	Case group	Control group	Confounders adjusted	NOS scores
Jazmine	2024	American	Case control study	International Statistical Classif cation of Diseases and Related Health Problems (ICD) or International Classification of Diseases for Oncology (ICD-O) topography codes (C54.0–C55.9 from ICD-10 for reference)	10,215	111,528	/	8
Britton	2020	American	Case control study	International Classification of Disease (ICD) topography codes (C54.0–55.9 from ICD-10 for reference)	10,924	123,749	Country and categorical birth year age at index date (continuous), marital status at first birth (unmarried, married/cohabiting, divorced/widowed, unknown/missing), pre- or early-pregnancy smoking	8
Anders	2019	Denmark	Cohort study	ICD-10 (international classification of diseases, 10th revision) codes C54-55 in combination with an endometrial cancer histological subtype by relevant ICD-O-3 (international classification of diseases for oncology, third edition) code	/	/	Age and time, pregnancy history, educational attainment, marital status, and urbanicity, induced abortion or birth, obesity and oral contraceptives use	8
Laure	2010	Germany	Cohort study	/	1,017	302,618	BMI, physical activity, alcohol, diabetes, smoking status and education	7
Ruth	2009	American	Case control study	ICD 7th Revision, code172	7,386	2,674,465(total number)	/	8
Marianne	2002	Finland	Cohort study	/	419	86,978(total number)	/	7
Fabio	1998	Italy	Case control study	/	588	2,089	Age, education, BMI, parity, oral contraceptive and hormonal replacement therapy use, age at menopause, diabetes, hypertension and smoking	7
Grethe	1995	Norway	Cohort study	ICD 7th Revision, code 172	428	9,307	Age	7

### Quality assessment

According to NOS criteria, the average score was 7.5 of all included studies, and the score for each trail was 7 or above, indicating that all studies were of high quality in this meta-analysis. The scores of the included studies are shown in Table 1 and S1 Table.

#### 0-10 years since last birth and risk of endometrial cancer.

**Fig 2:**
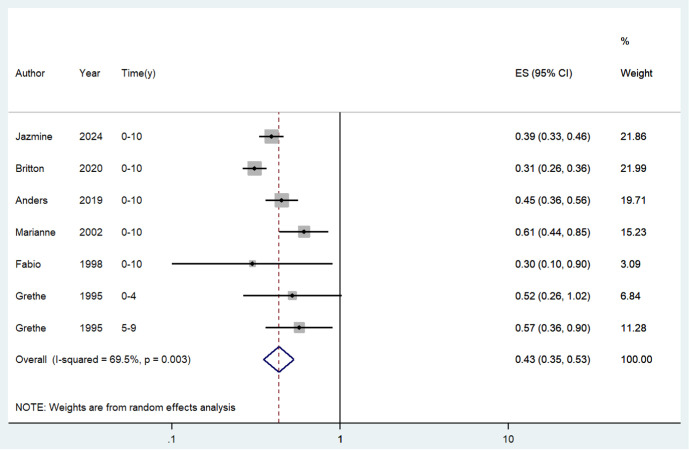
Meta-analysis of the risk of endometrial cancer of 0-10 years since last birth.

Seven included study states [19–22,24–26] assessed the association between 0–10 years since last birth and the risk of endometrial cancer. Overall, the history of 0–10 years since last birth was associated with a decreased risk of endometrial cancer (OR = 0.431; 95% CI: 0.351–0.530; I^2^ = 69.5%, P = 0.003; Fig 2). Sensitivity analysis showed that none of the individual studies reversed the pooled-effect size, which means that the results are robust (S1 Fig). Egger’s regression test (P = 0.000) likewise indicated no publication bias in our meta-analysis.

#### 10-20 years since last birth and Risk of endometrial cancer.

**Fig 3:**
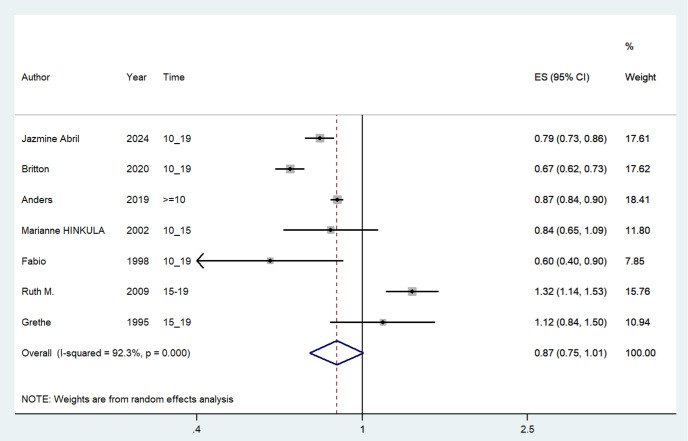
Meta-analysis of the risk of endometrial cancer of 10-20 years since last birth.

Seven included studies [19–21,23–26] assessed the association between 10–20 years since last birth and the risk of endometrial cancer. Overall, the history of 10–20 years since last birth was associated with a decreased risk of endometrial cancer (OR = 0.867; 95% CI: 0.747–1.007; I² = 78.03%, P = 0.000; Fig 3). Sensitivity analysis showed that none of the individual studies reversed the pooled-effect size, indicating robust results (S2 Fig). A visual inspection of the funnel plot showed no evidence of significant publication bias for the included studies on this outcome (Fig 5). Egger’s regression test (P = 0.062) similarly indicated no publication bias in our meta-analysis.

**Fig 4:**
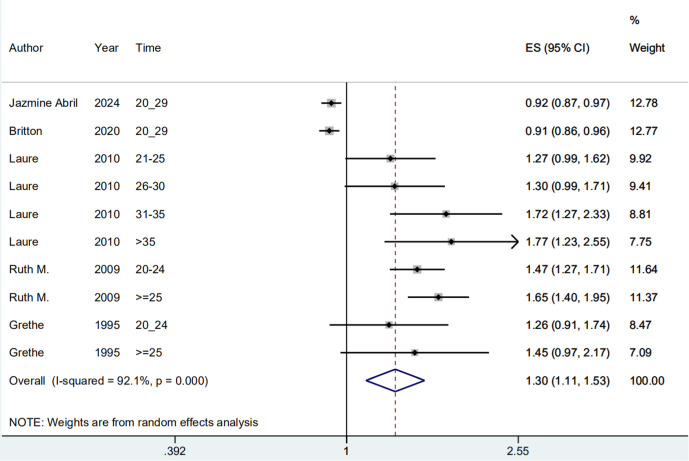
Meta-analysis of the risk of endometrial cancer of above 20 years since last birth.

**Fig 5:**
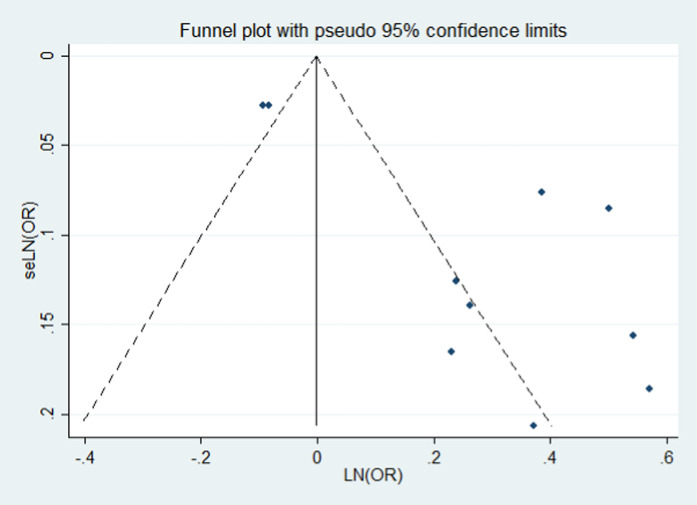
Publication bias of the risk of all groups.

#### Above 20 years *since* last birth and Risk *of* endometrial cancer.

Ten included study states [19–22,24–26] assessed the association between above 20 years since last birth and the risk of endometrial cancer. Overall, the history of above 20 years since last birth was associated with an increased risk of endometrial cancer (OR = 1.304; 95% CI: 1.111–1.530; I2 = 92.10%, P = 0.000; Fig 4). Sensitivity analysis showed that none of the individual studies had reversed the pooled-effect size, which means that the results are robust (S3 Fig).

#### Publication bias.

Visual examination of the funnel plot revealed no evidence of significant publication bias for the studies included in this meta-analysis (Fig 5). Additionally, Egger’s regression test (P = 0.062) confirmed the absence of publication bias.

## Discussion

### Main findings

This meta-analysis, encompassing 5 case-control studies and 3 cohort studies with a total of 3,310,734 participants, provides a comprehensive assessment of the relationship between time since last birth and endometrial cancer risk. We observed a significant reduction in endometrial cancer risk with 0–10 years and 10–20 years since the last birth, showing a 0.431-fold and 0.867-fold decrease in risk, respectively, compared to controls. Conversely, a time since last birth of over 20 years was associated with a 1.304-fold increase in risk. These findings suggest that time since last birth may be an independent risk factor for endometrial cancer.

### Interpretation of findings

Our analysis, which integrates data from both case-control and cohort studies, underscores the independent effect of time since last birth on endometrial cancer risk. Shorter intervals since the last birth appear to offer protective benefits to the endometrial lining, while longer intervals in multiparous women significantly elevate risk. To ensure the reliability of our conclusions, all included studies controlled for key confounding factors, which could potentially influence the association between reproductive history and endometrial cancer risk. Specifically:

Age: As a well-established risk factor, age was consistently adjusted for across all studies. Older age is associated with cumulative estrogen exposure and higher endometrial cancer risk, and thus adjusting for it reduces potential bias.

Parity (number of births): Multiparity may reduce the lifetime risk of endometrial cancer through cumulative hormonal and immune changes during pregnancy. By controlling for parity, our results specifically isolate the effect of time since last birth.

Metabolic comorbidities: Conditions such as obesity, diabetes, and hypertension are major risk factors for endometrial cancer, often mediated by increased estrogen levels. All studies adjusted for these variables to minimize their confounding effects.

Hormonal factors: Long-term estrogen use, hormone replacement therapy, and oral contraceptive use were also considered in most studies, as they can independently modify endometrial cancer risk.

Despite these adjustments, it is possible that some residual confounding exists, particularly from unmeasured factors such as genetic predisposition, lifestyle behaviors (diet, exercise), or environmental exposures. Future studies should aim to incorporate these variables for a more comprehensive risk assessment. Several pathophysiological mechanisms could explain the observed associations between time since last birth and endometrial cancer risk:

Progesterone’s Protective Effect: High levels of progesterone during pregnancy may help remove premalignant lesions or abnormal endometrial cells, reducing the risk of malignant transformation [27].

Mechanical Clearance: Childbirth and uterine involution may mechanically clear abnormal cells, offering protection against endometrial cancer in the years immediately following delivery.

Immunological and Hormonal Shifts: Pregnancy induces immune modulation and hormonal changes (e.g., increased placental hormones) that may influence endometrial health. However, the long-term implications of these changes remain underexplored [28].

Unopposed Estrogen Hypothesis: Prolonged exposure to high levels of estrogen without progesterone opposition can promote endometrial cell proliferation, increasing cancer risk [29]. Conversely, pregnancies closer to the time of diagnosis may limit this exposure through shorter intervals of unopposed estrogen [30].

Our findings suggest that shorter intervals since last pregnancy might be protective due to high progesterone levels during pregnancy clearing malignant cells. Conversely, longer intervals in multiparous women were associated with higher endometrial cancer risk, potentially due to prolonged unopposed estrogen exposure. Women who are older at their final birth may have reduced endometrial cancer risk, as sustaining pregnancy later in life may indicate a healthier endometrial environment or fewer anovulatory cycles.

### Implications and limitations

This meta-analysis highlights the importance of reproductive history as a factor in assessing endometrial cancer risk. Clinicians should consider time since last birth as part of a comprehensive risk evaluation, especially in multiparous women with extended intervals since their last delivery. Women in this category should be counseled on their increased risk and may benefit from regular gynecological examinations and screenings, including endometrial biopsy or transvaginal ultrasound, as part of a personalized prevention strategy.

While our analysis is based on robust data, several limitations should be acknowledged. The inclusion of both cohort and case-control studies introduces methodological heterogeneity, which, despite statistical adjustments, may still leave residual variability affecting the results. Additionally, unmeasured confounding variables, such as genetic predisposition, lifestyle factors (e.g., smoking, diet), and socioeconomic status, were not consistently reported or adjusted for across studies. Cross-sectional studies were excluded to strengthen causal inference, but this decision may have narrowed the scope of data and limited the breadth of our findings.

Future research should incorporate a broader range of study designs, including cross-sectional studies, to validate findings across diverse populations. Greater exploration of additional covariates, such as genetic and lifestyle factors, is necessary to fully elucidate the complex relationship between reproductive history and endometrial cancer risk. Investigating the biological mechanisms underlying the protective effects of pregnancy and the increased risk associated with prolonged intervals since last birth will provide more comprehensive insights into preventive strategies.

## Supporting information

S1 Fig Sensitivity analysis of the risk of endometrial cancer of 0–10 years since last birth. (DOCX)

S2 Fig Sensitivity analysis of the risk of endometrial cancer of 10–20 years since last birth. (DOCX)

S3 Fig Sensitivity analysis of the risk of endometrial cancer of above 20 years since last birth. (DOCX)

S1 Table The quality assessment of cohort and case-control studies. (DOCX)

S2 Table The numble table of all studies identified in the literature search. (DOCX)

S3 Table All data extracted from the primary research sources. (DOCX)

S1 File Search strategy in different databases. (DOCX)

S1 PRISMA_2020 Checklist. (DOCX)

## References

[1] Żyła MM, Wilczyński JR, Kostrzewa M, Księżakowska-Łakoma K, Nowak M, Stachowiak G, et al. The significance of markers in the diagnosis of endometrial cancer. Prz Menopauzalny. 2016;15(3):176–85. doi: 10.5114/pm.2016.63500 27980530 PMC5137482

[2] Zhang L, Wan Y, Jiang Y, Ma J, Liu J, Tang W, et al. Upregulation HOXA10 homeobox gene in endometrial cancer: role in cell cycle regulation. Med Oncol. 2014;31(7):52. doi: 10.1007/s12032-014-0052-2 24943991

[3] Trabert B, Eldridge RC, Pfeiffer RM, Shiels MS, Kemp TJ, Guillemette C, et al. Prediagnostic circulating inflammation markers and endometrial cancer risk in the prostate, lung, colorectal and ovarian cancer (PLCO) screening trial. Int J Cancer. 2017;140(3):600–10. doi: 10.1002/ijc.30478 27770434 PMC5159268

[4] Setiawan VW, Yang HP, Pike MC, McCann SE, Yu H, Xiang Y-B, et al. Type I and II endometrial cancers: have they different risk factors?. J Clin Oncol. 2013;31(20):2607–18. doi: 10.1200/JCO.2012.48.2596 23733771 PMC3699726

[5] Hallum S, Pinborg A, Kamper-Jørgensen M. Long-term impact of preeclampsia on maternal endometrial cancer risk. Br J Cancer. 2016;114(7):809–12. doi: 10.1038/bjc.2016.55 26964032 PMC4984869

[6] Burton GJ, Jauniaux E. The cytotrophoblastic shell and complications of pregnancy. Placenta. 2017;60:134–9. doi: 10.1016/j.placenta.2017.06.007 28651899

[7] Pocobelli G, Doherty JA, Voigt LF, Beresford SA, Hill DA, Chen C, et al. Pregnancy history and risk of endometrial cancer. Epidemiology. 2011;22(5):638–45. doi: 10.1097/EDE.0b013e3182263018 21691206 PMC3152311

[8] Jordan SJ, Na R, Weiderpass E, Adami H-O, Anderson KE, van den Brandt PA, et al. Pregnancy outcomes and risk of endometrial cancer: A pooled analysis of individual participant data in the Epidemiology of Endometrial Cancer Consortium. Int J Cancer. 2021;148(9):2068–78. doi: 10.1002/ijc.33360 33105052 PMC7969437

[9] Żyła MM, Kostrzewa M, Litwińska E, Szpakowski A, Wilczyński JR, Stetkiewicz T. The role of angiogenic factors in endometrial cancer. Prz Menopauzalny. 2014;13(2):122–6. doi: 10.5114/pm.2014.42714 26327841 PMC4520350

[10] Chatzaki E, Bax CM, Eidne KA, Anderson L, Grudzinskas JG, Gallagher CJ. The expression of gonadotropin-releasing hormone and its receptor in endometrial cancer, and its relevance as an autocrine growth factor. Cancer Res. 1996;56(9):2059–65. 8616851

[11] Coenegrachts L, Schrauwen S, Van Bree R, Despierre E, Luyten C, Jonckx B, et al. Increased expression of placental growth factor in high-grade endometrial carcinoma. Oncol Rep. 2013;29(2):413–8. doi: 10.3892/or.2012.2178 23232836 PMC3583572

[12] Kaaks R, Lukanova A, Kurzer MS. Obesity, endogenous hormones, and endometrial cancer risk: a synthetic review. Cancer Epidemiol Biomarkers Prev. 2002;11(12):1531–43. 12496040

[13] Devor EJ, Reyes HD, Santillan DA, Santillan MK, Onukwugha C, Goodheart MJ, et al. Placenta-specific protein 1: a potential key to many oncofetal-placental OB/GYN research questions. Obstet Gynecol Int. 2014;2014:678984. doi: 10.1155/2014/678984 24757447 PMC3976915

[14] Sonderegger S, Pollheimer J, Knöfler M. Wnt signalling in implantation, decidualisation and placental differentiation--review. Placenta. 2010;31(10):839–47. doi: 10.1016/j.placenta.2010.07.011 20716463 PMC2963059

[15] Taylor HS, Arici A, Olive D, Igarashi P. HOXA10 is expressed in response to sex steroids at the time of implantation in the human endometrium. J Clin Invest. 1998;101(7):1379–84. doi: 10.1172/JCI1057 9525980 PMC508715

[16] Page MJ, McKenzie JE, Bossuyt PM, Boutron I, Hoffmann TC, Mulrow CD, et al. The PRISMA 2020 statement: an updated guideline for reporting systematic reviews. BMJ. 2021;372:n71. doi: 10.1136/bmj.n71 33782057 PMC8005924

[17] Li X, Huang L, Tang Y, Hu X, Wen C. Gout and risk of dementia, Alzheimer’s disease or vascular dementia: a meta-epidemiology study. Front Aging Neurosci. 2023;15:1051809. doi: 10.3389/fnagi.2023.1051809 37181628 PMC10169719

[18] Xie W, Wang Y, Xiao S, Qiu L, Yu Y, Zhang Z. Association of gestational diabetes mellitus with overall and type specific cardiovascular and cerebrovascular diseases: systematic review and meta-analysis. BMJ. 2022;378:e070244. doi: 10.1136/bmj-2022-070244 36130740 PMC9490552

[19] Albrektsen G, Heuch I, Tretli S, Kvåle G. Is the risk of cancer of the corpus uteri reduced by a recent pregnancy? A prospective study of 765,756 Norwegian women. Int J Cancer. 1995;61(4):485–90. doi: 10.1002/ijc.2910610410 7759154

[20] Parazzini F, Negri E, La Vecchia C, Benzi G, Chiaffarino F, Polatti A, et al. Role of reproductive factors on the risk of endometrial cancer. Int J Cancer. 1998;76(6):784–6. doi: 10.1002/(sici)1097-0215(19980610)76:6<784::aid-ijc2>3.0.co;2-u 9626340

[21] Pfeiffer RM, Mitani A, Landgren O, Ekbom A, Kristinsson SY, Björkholm M, et al. Timing of births and endometrial cancer risk in Swedish women. Cancer Causes Control. 2009;20(8):1441–9. doi: 10.1007/s10552-009-9370-7 19565342 PMC2843814

[22] Dossus L, Allen N, Kaaks R, Bakken K, Lund E, Tjonneland A, et al. Reproductive risk factors and endometrial cancer: the European Prospective Investigation into Cancer and Nutrition. Int J Cancer. 2010;127(2):442–51. doi: 10.1002/ijc.25050 19924816

[23] Hinkula M, Pukkala E, Kyyrönen P, Kauppila A. Grand multiparity and incidence of endometrial cancer: a population-based study in Finland. Int J Cancer. 2002;98(6):912–5. doi: 10.1002/ijc.10267 11948472

[24] Husby A, Wohlfahrt J, Melbye M. Pregnancy duration and endometrial cancer risk: nationwide cohort study. BMJ. 2019;366:l4693. doi: 10.1136/bmj.l4693 31412996 PMC6693051

[25] Trabert B, Troisi R, Grotmol T, Ekbom A, Engeland A, Gissler M, et al. Associations of pregnancy-related factors and birth characteristics with risk of endometrial cancer: A Nordic population-based case-control study. Int J Cancer. 2020;146(6):1523–31. doi: 10.1002/ijc.32494 31173648 PMC6898733

[26] Abril J, Trabert B, Troisi R, Grotmol T, Ekbom A, Engeland A, et al. Associations between pregnancy-related factors and birth characteristics with risk of rare uterine cancer subtypes: a Nordic population-based case-control study. Cancer Causes Control. 2024;35(5):741–7. doi: 10.1007/s10552-023-01832-6 38129544 PMC11441445

[27] Ursin G, Palla SL, Reboussin BA, Slone S, Wasilauskas C, Pike MC, et al. Post-treatment change in serum estrone predicts mammographic percent density changes in women who received combination estrogen and progestin in the Postmenopausal Estrogen/Progestin Interventions (PEPI) Trial. J Clin Oncol. 2004;22(14):2842–8. doi: 10.1200/JCO.2004.03.120 15254051

[28] Sköld C, Bjørge T, Ekbom A, Engeland A, Gissler M, Grotmol T, et al. Preterm delivery is associated with an increased risk of epithelial ovarian cancer among parous women. Int J Cancer. 2018;143(8):1858–67. doi: 10.1002/ijc.31581 29737528 PMC6128744

[29] Key TJ, Pike MC. The dose-effect relationship between “unopposed” oestrogens and endometrial mitotic rate: its central role in explaining and predicting endometrial cancer risk. Br J Cancer. 1988;57(2):205–12. doi: 10.1038/bjc.1988.44 3358913 PMC2246441

[30] Key TJ, Verkasalo PK, Banks E. Epidemiology of breast cancer. Lancet Oncol. 2001;2(3):133–40. doi: 10.1016/S1470-2045(00)00254-0 11902563

