# Supplementary figures and images for: Time since last birth and the risk of endometrial cancer: A meta-analysis of observational studies
Source: PLoS One. 2025 Jul 8;20(7):e0325907. doi: 10.1371/journal.pone.0325907 (PMC12237066; doi:10.1371/journal.pone.0325907)

**S1 Fig Sensitivity analysis of the risk of endometrial cancer of 0-10 years since last birth**


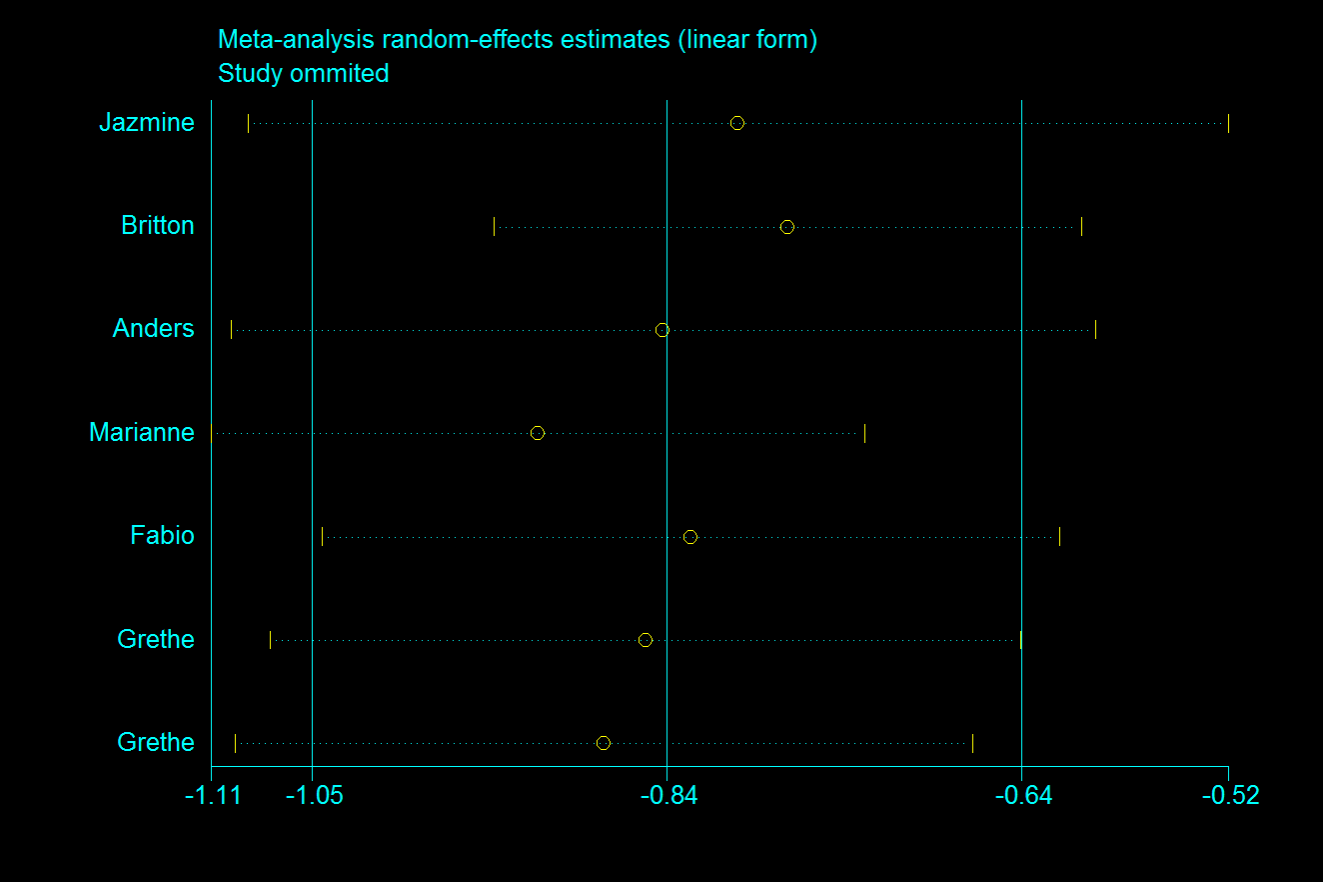

Supplement: S1 Fig — (DOCX) [file pone.0325907.s001.docx]

**S2 Fig Sensitivity analysis of the risk of endometrial cancer of 10-20 years since last birth**


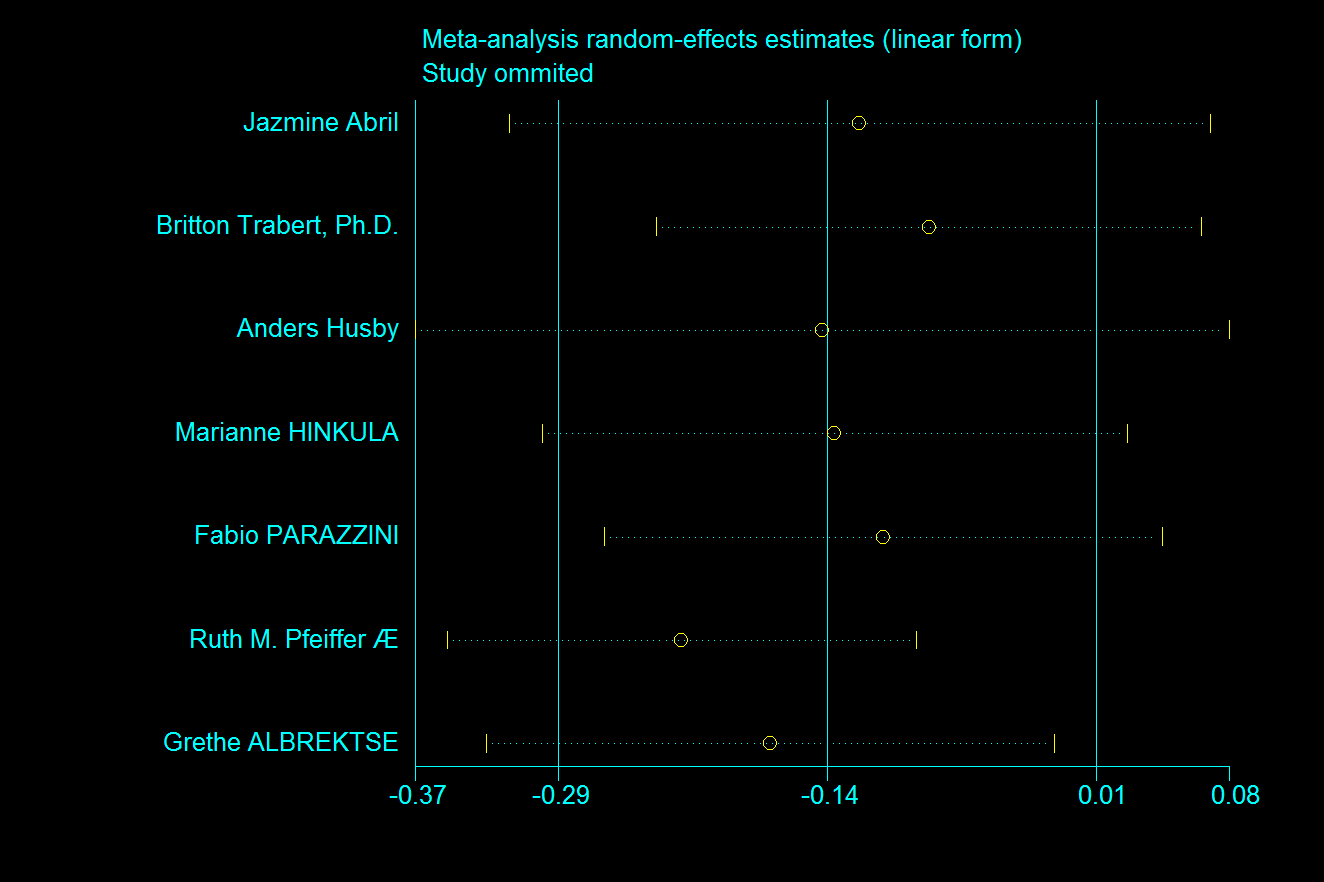

Supplement: S2 Fig — (DOCX) [file pone.0325907.s002.docx]

**S3 Fig Sensitivity analysis of the risk of endometrial cancer of above 20 years since last birth**


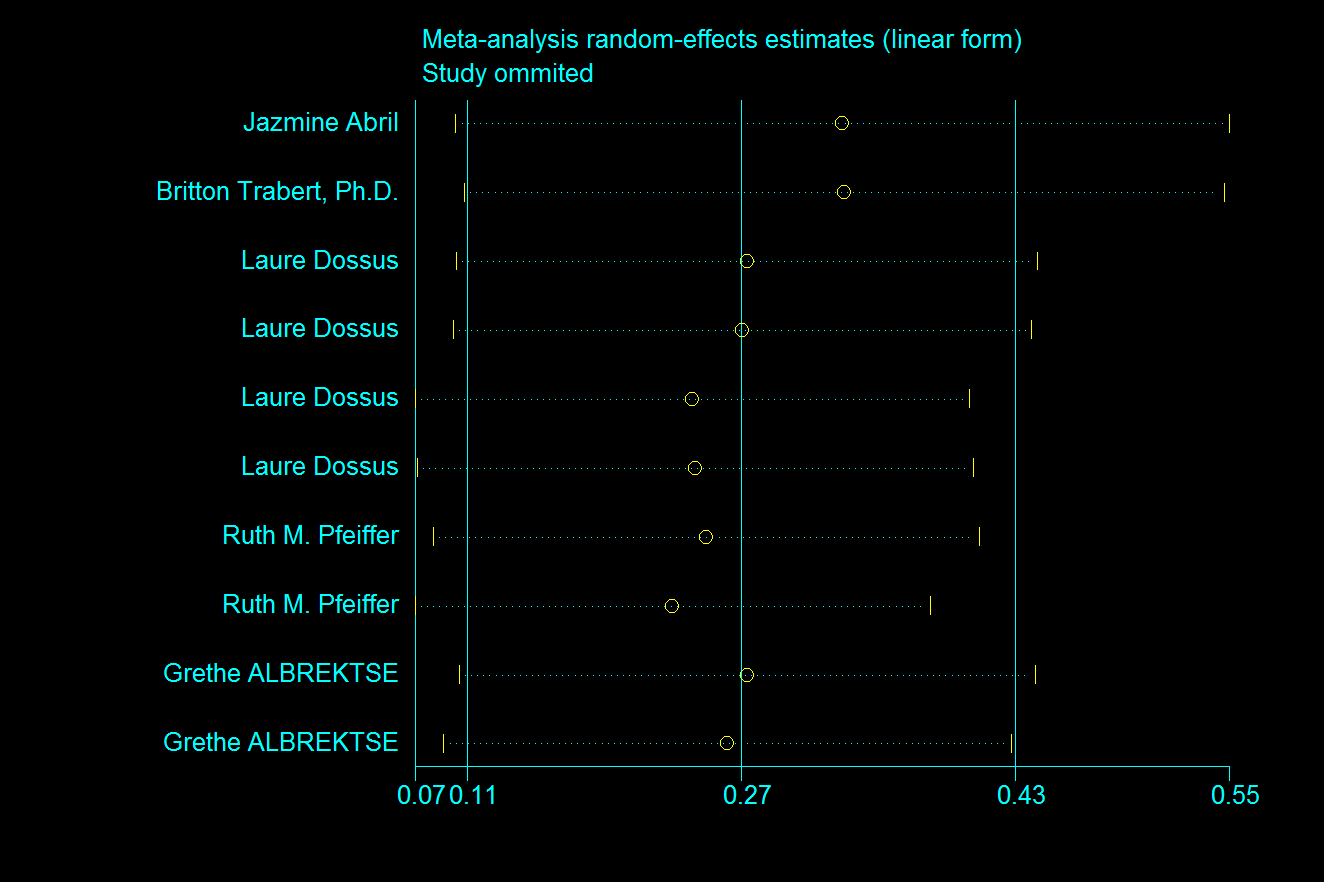

Supplement: S3 Fig — (DOCX) [file pone.0325907.s003.docx]
