# Supplementary material for: Time since last birth and the risk of endometrial cancer: A meta-analysis of observational studies
Source: PLoS One. 2025 Jul 8;20(7):e0325907. doi: 10.1371/journal.pone.0325907 (PMC12237066; doi:10.1371/journal.pone.0325907)
Supplement: S1 Table — (DOCX) [file pone.0325907.s004.docx]

**S1 Table The quality assessment of cohort and case-control studies.**

| **Study** | **Year** | **Selection** | **Comparability** | **Outcome** | **Total** |
| --- | --- | --- | --- | --- | --- |
| Cohort studies (n=3) | | | | | |
| Anders Husby | 2019 | *** | ** | *** | 8 |
| Laure Dossus | 2009 | ** | * | ** | 7 |
| Grethe Albrektsen | 1995 | *** | ** | ** | 7 |
| Case-control studies (n=5) | | | | | |
| Jazmine Abril | 2024 | *** | ** | *** | 8 |
| Britton Trabert | 2020 | *** | ** | *** | 8 |
| Ruth M.Pfeiffer | 2008 | **** | ** | ** | 7 |
| Marianne Hinkula | 2002 | *** | ** | ** | 7 |
| Fabio Parazzini | 1998 | *** | ** | *** | 8 |

The NOS scale was used to evaluate the quality of the cohort and case-control studies
