# Supplementary material for: Time since last birth and the risk of endometrial cancer: A meta-analysis of observational studies
Source: PLoS One. 2025 Jul 8;20(7):e0325907. doi: 10.1371/journal.pone.0325907 (PMC12237066; doi:10.1371/journal.pone.0325907)
Supplement: S2 Table — (DOCX) [file pone.0325907.s005.docx]

**S2 Table The numble table of all studies identified in the literature search**

| **No.** | **Title** | **Author** | **Year** | **Journal** | **Included** | **Reason** |
| --- | --- | --- | --- | --- | --- | --- |
| 1 | Associations between pregnancy-related factors and birth characteristics with risk of rare uterine cancer subtypes:a Nordicpopulation-based case-control study | Jazmine,et al | 2024 | Cance Causes& Control | Yes | — |
| 2 | The role of pregnancy in maternal cancer risk: Epidemiologic evidence from the Nordic Countries Linked Birth and Cancer Registries Cohort Project. | Troisi R,et al | 2022 | Norsk Epidemiologi | No | Wrong publication type |
| 3 | Complications of Pregnancy and the Risk of Developing Endometrial or Ovarian Cancer: A Case-Control Study. | Liu Y,et al | 2021 | Frontiers in Endocrinology | No | Irrelevant topic |
| 4 | Endometrial cancer risk after fertility treatment: a population-based cohort study. | Guleria S,et al | 2021 | Cancer Causes& Control | No | Irrelevant topic |
| 5 | Pregnancy outcomes and risk of endometrial cancer: A pooled analysis of individual participant data in the Epidemiology of Endometrial Cancer Consortium. | Jordan S,et al | 2021 | International Journal of Cancer | No | Irrelevant topic |
| 6 | Associations of pregnancy-related factors and birth characteristics with risk of endometrial cancer: A Nordic population-based case–control study. | Britton, et al | 2020 | International Journal of Cancer | Yes | — |
| 7 | Pregnancy duration and endometrial cancer risk: Nationwide cohort study. | Anders,et al | 2019 | The BMJ | Yes | — |
| 8 | Long-term effect of pregnancy-related factors on the development of endometrial neoplasia: A nationwide retrospective cohort study. | Cho, H,et al | 2019 | PLoS One | No | Irrelevant topic |
| 9 | Age at first birth and the risk of endometrial cancer incidence: A pooled analysis of two prospective cohort studies among Japanese women. | Sugawara Y,et al | 2018 | Journal of Cancer | No | Irrelevant topic |
| 10 | Infertility and incident endometrial cancer risk: a pooled analysis from the epidemiology of endometrial cancer consortium (E2C2). | Yang H,et al | 2015 | Br J Cancer | No | Irrelevant topic |
| 11 | Reproductive risk factors and endometrial cancer: The European prospective investigation into cancer and nutrition. | Laure,et al | 2010 | International Journal of Cancer | Yes | — |
| 12 | Timing of births and endometrial cancer risk in Swedish women. | Ruth,et al | 2009 | Cancer Causes & Control | Yes | — |
| 13 | Parity and time interval since childbirth influence survival in endometrial cancer patients. | Albrektsen G,et al | 2009 | International Journal of Gynecological Cancer | No | Wrong syudy design |
| 14 | Lactation and risk of endometrial cancer in Japan: A case-control study. | Okamura C,et al | 2006 | Tohoku Journal of Experimental Medicine | No | Irrelevant topic |
| 15 | Menstrual and reproductive factors in relation to risk of endometrial cancer in Chinese women. | Wernli K.J,et al | 2006 | Cancer Causes& Control | No | Insufficient data |
| 16 | Menstrual and reproductive factors and endometrial cancer risk: Results from a population-based case-control study in urban shanghai. | Xu W. H,et al | 2004 | International Journal of Cancer | No | Insufficient data |
| 17 | Grand multiparity and incidence of endometrial cancer: A population-based study in Finland. | Marianne,et al | 2002 | International Journal of Cancer | Yes | — |
| 18 | The effect of nulliparity on survival in endometrial cancer at different ages. | Hachisuga T,et al | 2001 | Gynecol Oncol | No | Wrong syudy design |
| 19 | Role of reproductive factors on the risk of endometrial cancer. | Fabio,et al | 1998 | International Journal of Cancer | Yes | — |
| 20 | Reproductive factors and risk of endometrial cancer: The Iowa Women's Health study. | McPherson C P,et al | 1996 | American Journal of Epidemiology | No | Insufficient data |
| 21 | Is the risk of cancer of the corpus uteri reduced by a recent pregnancy? A prospective study of 765,756 Norwegian women. | Grethe,et al | 1995 | International Journal of Cancer | Yes | — |
