# Supplementary material for: Time since last birth and the risk of endometrial cancer: A meta-analysis of observational studies
Source: PLoS One. 2025 Jul 8;20(7):e0325907. doi: 10.1371/journal.pone.0325907 (PMC12237066; doi:10.1371/journal.pone.0325907)
Supplement: S3 Table — (DOCX) [file pone.0325907.s006.docx]

**S3 Table All data extracted from the primary research sources**

| **Author** | **Year** | **Time(y)** | **OR** | **OR-LL** | **OR-UL** | **LN(OR)** | **seLN(OR)** |
| --- | --- | --- | --- | --- | --- | --- | --- |
| Jazmine | 2024 | 0-10 | 0.39 | 0.33 | 0.46 | -0.94160854 | 0.084728019 |
| Britton | 2020 | 0-10 | 0.31 | 0.26 | 0.36 | -1.171182982 | 0.083015918 |
| Anders | 2019 | 0-10 | 0.45 | 0.36 | 0.56 | -0.798507696 | 0.112712437 |
| Marianne | 2002 | 0-10 | 0.61 | 0.44 | 0.86 | -0.494296322 | 0.170958587 |
| Fabio | 1998 | 0-10 | 0.3 | 0.1 | 0.9 | -1.203972804 | 0.560516474 |
| Grethe | 1995 | 0-4 | 0.52 | 0.27 | 1.04 | -0.653926467 | 0.344018886 |
| Grethe | 1995 | 5-9 | 0.57 | 0.36 | 0.90 | -0.562118918 | 0.233747636 |
| Jazmine | 2024 | 10_19 | 0.79 | 0.73 | 0.86 | -0.235722334 | 0.041808126 |
| Britton | 2020 | 10_19 | 0.67 | 0.62 | 0.73 | -0.400477567 | 0.041664555 |
| Anders | 2019 | >=10 | 0.87 | 0.84 | 0.90 | -0.139262067 | 0.017600222 |
| Marianne | 2002 | 10_15 | 0.84 | 0.65 | 1.1 | -0.174353387 | 0.134207422 |
| Fabio | 1998 | 10_19 | 0.6 | 0.4 | 0.9 | -0.510825624 | 0.206869953 |
| Ruth | 2009 | 15-19 | 1.32 | 1.14 | 1.53 | 0.277631737 | 0.07506109 |
| Grethe | 1995 | 15_19 | 1.12 | 0.84 | 1.5 | 0.113328685 | 0.147912881 |
| Jazmine | 2024 | 20_29 | 0.92 | 0.87 | 0.97 | -0.083381609 | 0.027755832 |
| Britton | 2020 | 20_29 | 0.91 | 0.86 | 0.96 | -0.094310679 | 0.028061453 |
| Laure | 2010 | 21-25 | 1.27 | 0.99 | 1.62 | 0.2390169 | 0.125631756 |
| Laure | 2010 | 26-30 | 1.3 | 0.99 | 1.71 | 0.262364264 | 0.139424415 |
| Laure | 2010 | 31-35 | 1.72 | 1.27 | 2.34 | 0.542324291 | 0.155901538 |
| Laure | 2010 | >35 | 1.77 | 1.23 | 2.55 | 0.570979547 | 0.185989589 |
| Ruth | 2009 | 20-24 | 1.47 | 1.26 | 1.7 | 0.385262401 | 0.076407278 |
| Ruth | 2009 | >=25 | 1.65 | 1.39 | 1.94 | 0.500775288 | 0.085046996 |
| Grethe | 1995 | 20_24 | 1.26 | 0.91 | 1.74 | 0.231111721 | 0.16535607 |
| Grethe | 1995 | >=25 | 1.45 | 0.97 | 2.18 | 0.371563556 | 0.206577573 |
