# Supplementary material for: Time since last birth and the risk of endometrial cancer: A meta-analysis of observational studies
Source: PLoS One. 2025 Jul 8;20(7):e0325907. doi: 10.1371/journal.pone.0325907 (PMC12237066; doi:10.1371/journal.pone.0325907)
Supplement: S1 File — (DOCX) [file pone.0325907.s007.docx]

**S1 File Search strategy in different databases.**

**PubMed**

| **No.** | **Content** | **Result** |
| --- | --- | --- |
| #1 | Search: "Pregnancy"[Mesh] Sort by: Most Recent | 1030979 |
| #2 | Search: ((Pregnanc*[Title/Abstract]) OR (Gestation[Title/Abstract])) OR (Birth[Title/Abstract]) | 859720 |
| #3 | Search: ("Pregnancy"[Mesh]) OR (((Pregnanc*[Title/Abstract]) OR (Gestation[Title/Abstract])) OR (Birth[Title/Abstract])) | 1364416 |
| #4 | Search: "Endometrial Neoplasms"[Mesh] Sort by: Most Recent | 26920 |
| #5 | Search: ((((((("Endometrial Neoplasm*"[Title/Abstract]) OR ("Endometrial Carcinoma*"[Title/Abstract])) OR ("Endometrial Cancer*"[Title/Abstract])) OR ("Endometrium Cancer*"[Title/Abstract])) OR ("Cancer of the Endometrium"[Title/Abstract])) OR ("Carcinoma of Endometrium"[Title/Abstract])) OR ("Endometrium Carcinoma*"[Title/Abstract])) OR ("Cancer of Endometrium"[Title/Abstract]) | 32405 |
| #6 | Search: ("Endometrial Neoplasms"[Mesh]) OR (((((((("Endometrial Neoplasm*"[Title/Abstract]) OR ("Endometrial Carcinoma*"[Title/Abstract])) OR ("Endometrial Cancer*"[Title/Abstract])) OR ("Endometrium Cancer*"[Title/Abstract])) OR ("Cancer of the Endometrium"[Title/Abstract])) OR ("Carcinoma of Endometrium"[Title/Abstract])) OR ("Endometrium Carcinoma*"[Title/Abstract])) OR ("Cancer of Endometrium"[Title/Abstract])) | 40239 |
| #7 | Search: (("Pregnancy"[Mesh]) OR (((Pregnanc*[Title/Abstract]) OR (Gestation[Title/Abstract])) OR (Birth[Title/Abstract]))) AND (("Endometrial Neoplasms"[Mesh]) OR (((((((("Endometrial Neoplasm*"[Title/Abstract]) OR ("Endometrial Carcinoma*"[Title/Abstract])) OR ("Endometrial Cancer*"[Title/Abstract])) OR ("Endometrium Cancer*"[Title/Abstract])) OR ("Cancer of the Endometrium"[Title/Abstract])) OR ("Carcinoma of Endometrium"[Title/Abstract])) OR ("Endometrium Carcinoma*"[Title/Abstract])) OR ("Cancer of Endometrium"[Title/Abstract]))) | 1867 |
| #8 | Search: "Risk"[Mesh] Sort by: Most Recent | 1412524 |
| #9 | Search: risk[Title/Abstract] Sort by: Most Recent | 2919483 |
| #10 | Search: ("Risk"[Mesh]) OR (risk[Title/Abstract]) Sort by: Most Recent | 3441145 |
| #11 | Search: (("Risk"[Mesh]) OR (risk[Title/Abstract])) AND ((("Pregnancy"[Mesh]) OR (((Pregnanc*[Title/Abstract]) OR (Gestation[Title/Abstract])) OR (Birth[Title/Abstract]))) AND (("Endometrial Neoplasms"[Mesh]) OR (((((((("Endometrial Neoplasm*"[Title/Abstract]) OR ("Endometrial Carcinoma*"[Title/Abstract])) OR ("Endometrial Cancer*"[Title/Abstract])) OR ("Endometrium Cancer*"[Title/Abstract])) OR ("Cancer of the Endometrium"[Title/Abstract])) OR ("Carcinoma of Endometrium"[Title/Abstract])) OR ("Endometrium Carcinoma*"[Title/Abstract])) OR ("Cancer of Endometrium"[Title/Abstract])))) Sort by: Most Recent | 805 |

**Embase**

| **No.** | **Content** | **Result** |
| --- | --- | --- |
| #1 | 'pregnancy'/exp | 907494 |
| #2 | pregnanc*:ab,ti OR gestation:ab,ti OR birth:ab,ti | 1112098 |
| #3 | #1 OR #2 | 1473240 |
| #4 | 'risk'/exp | 3233526 |
| #5 | risk:ab,ti | 4198982 |
| #6 | #4 OR #5 | 5147411 |
| #7 | 'endometrium tumor'/exp | 89568 |
| #8 | 'endometrial carcinoma*':ab,ti OR 'endometrial cancer*':ab,ti OR 'endometrium cancer*':ab,ti OR 'cancer of the endometrium':ab,ti OR 'carcinoma of endometrium':ab,ti OR 'endometrium carcinoma*':ab,ti OR 'cancer of endometrium':ab,ti OR 'endometrial neoplasm*':ab,ti | 48600 |
| #9 | #6 OR #7 | 94672 |
| #10 | #3 AND #6 AND #9 | 2683 |

**Cochran Library**

| **No.** | **Content** | **Result** |
| --- | --- | --- |
| #1 | MeSH descriptor: [Pregnancy] explode all trees | 34005 |
| #2 | (Pregnanc*):ti,ab,kw OR (Gestation):ti,ab,kw OR (Birth):ti,ab,kw | 105266 |
| #3 | #1 OR #2 | 105477 |
| #4 | MeSH descriptor: [Endometrial Neoplasms] explode all trees | 1207 |
| #5 | (Endometrial Neoplasm*):ti,ab,kw OR (Endometrial Carcinoma*):ti,ab,kw OR (Endometrial Cancer*):ti,ab,kw OR (Endometrium Cancer*):ti,ab,kw OR (Cancer of the Endometrium):ti,ab,kw (Word variations have been searched) | 3418 |
| #6 | (Carcinoma of Endometrium):ti,ab,kw OR (Endometrium Carcinoma*):ti,ab,kw OR (Cancer of Endometrium):ti,ab,kw | 1590 |
| #7 | #4 OR #5 OR #6 | 3499 |
| #8 | MeSH descriptor: [Risk] explode all trees | 56374 |
| #9 | (risk):ti,ab,kw | 315513 |
| #10 | #8 OR #9 | 319420 |
| #11 | #3 AND #7 AND #10 | 51 |

**Web of science**

| **No.** | **Content** | **Result** |
| --- | --- | --- |
| #1 | ((TS=(Pregnanc*)) OR TS=(Gestation)) OR TS=(Birth) | 988454 |
| #2 | (((((((TS=(“Endometrial Neoplasm*” )) OR TS=(“Endometrial Carcinoma*”)) OR TS=(“Endometrial Cancer*”)) OR TS=(“Endometrium Cancer*”)) OR TS=(“Cancer of the Endometrium”)) OR TS=(“Carcinoma of Endometrium”)) OR TS=(“Endometrium Carcinoma*”)) OR TS=(“Cancer of Endometrium”) | 42388 |
| #3 | TS=(risk) | 4475432 |
| #4 | #1 AND #2 AND #3 | 547 |
